# Serum Iron Concentration, but Not Hemoglobin, Correlates with TIMI Risk Score and 6-Month Left Ventricular Performance after Primary Angioplasty for Acute Myocardial Infarction

**DOI:** 10.1371/journal.pone.0104495

**Published:** 2014-08-06

**Authors:** Ching-Hui Huang, Chia-Chu Chang, Chen-Ling Kuo, Ching-Shan Huang, Tzai-Wen Chiu, Chih-Sheng Lin, Chin-San Liu

**Affiliations:** 1 Division of Cardiology, Department of Internal Medicine, Changhua Christian Hospital, Changhua, Taiwan; 2 Department of Biological Science and Technology, National Chiao Tung University, Hsinchu, Taiwan; 3 Division of Nephrology, Department of Internal Medicine, Changhua Christian Hospital, Changhua, Taiwan; 4 School of Medicine, Chung Shan Medical University, Taichung, Taiwan; 5 Vascular and Genomic Research Center, Changhua Christian Hospital, Changhua, Taiwan; 6 Department of Neurology, Changhua Christian Hospital, Changhua, Taiwan; 7 Graduate Institute of Integrative Medicine, China Medical University, Taichung, Taiwan; University of Louisville, United States of America

## Abstract

**Objective:**

Anemia is associated with high mortality and poor prognosis after acute coronary syndrome (ACS). Increased red cell distribution width (RDW) is a strong independent predictor for adverse outcomes in ACS. The common underlying mechanism for anemia and increased RDW value is iron deficiency. It is not clear whether serum iron deficiency without anemia affects left ventricular (LV) performance after primary angioplasty for acute myocardial infarction (AMI). We investigated the prognostic value of serum iron concentration on LV ejection fraction (EF) at 6 months and its relationship to thrombolysis in myocardial infarction (TIMI) risk score in post MI patients.

**Methods:**

We recruited 55 patients who were scheduled to undergo primary coronary balloon angioplasty after AMI and 54 age- and sex-matched volunteers. Serum iron concentration and interleukin-6 levels were measured before primary angioplasty. LVEF was measured by echocardiography at baseline and after 6 months. TIMI risk score was calculated for risk stratification.

**Results:**

Serum iron concentration was significantly lower in those in whom LVEF had not improved ≥10% from baseline (52.7±24.1 *versus* 80.8±50.8 µg/dl, *P* = 0.016) regardless of hemoglobin level, and was significantly lower in the AMI group than in the control group (62.5±37.7 *versus* 103.0±38.1 µg/dl, *P*<0.001). Trend analysis revealed that serum iron concentration decreased as TIMI risk score increased (*P* = 0.002). In addition, lower serum iron concentrations were associated with higher levels of inflammatory markers. Multiple linear regression showed that baseline serum iron concentration can predict LV systolic function 6 months after primary angioplasty for AMI even after adjusting for traditional prognostic factors.

**Conclusion:**

Hypoferremia is not only a marker of inflammation but also a potential prognostic factor for LV systolic function after revascularization therapy for AMI, and may be a novel biomarker for therapeutic intervention.

## Introduction

Functional iron deficiency is associated with impaired left ventricular (LV) performance in patients with chronic heart failure (CHF) [1]. Treatment with intravenous iron can improve the symptoms of CHF in patients with reduced LV ejection fraction even in the absence of anemia [2]. In heart failure, systemic and myocardial iron stores are depleted [3], which raises the possibility that iron deficiency rather than lack of hemoglobin (Hb) might be of primary concern in heart failure [4]. Red cell distribution width (RDW) represents the variability in the size of circulating erythrocytes [5]. Most recently, increased RDW was shown to be a strong independent predictor of increased morbidity and mortality in patients with acute coronary syndrome even in nonanemic patients [6]–[8]. The common underlying mechanism for anemia and increased RDW value is iron deficiency. RDW values are increased in iron deficiency anemia. Iron deficiency is a common cause of anemia; nevertheless Hb concentration may be normal in the presence of iron deficiency. Iron is essential to many biological processes, particularly in mitochondria, where it catalyzes enzymatic reactions and is involved in the regulation of oxidative stress. Heart tissue is rich in mitochondria, making iron of particular importance to cardiac function [9]. Several strands of evidence have shown that anemic patients have a worse outcome after acute myocardial infarction (AMI) [10]–[12]. Few studies, however, have fully explored whether iron status changes during the course of AMI, and whether serum iron concentration affects LV performance after primary angioplasty.

Longitudinal studies have demonstrated that Interleukin 6 (IL-6) is a better predictor of CHF after acute coronary syndrome (ACS) than C-reactive protein (CRP) [13], and that it is also an independent predictor of cardiovascular mortality [14]. To the best of our knowledge, however, no studies have analyzed the relation between IL-6 concentration and serum iron concentration in patients with acute ST-segment elevation myocardial infarction (STEMI).

In this study, we examined whether baseline serum iron concentration measured at the time of primary angioplasty is predictive of LV function 6 months after STEMI. We also examined whether there were associations between serum iron concentration and factors traditionally associated with outcome after myocardial infarction, such as the concentrations of circulating inflammatory cytokines and the thrombolysis in myocardial infarction (TIMI) risk score, a validated clinical risk score that predicts short- and long-term mortality after STEMI [15], [16].

## Methods

### Subjects and study protocol

In this prospective study we enrolled 55 consecutive patients with *de* *novo* acute STEMI who underwent primary PCI and thromboaspiration between January 2010 and January 2011. We also enrolled a control group of 54 healthy age- and sex-matched volunteers to compare changes in serum iron and inflammatory cytokine levels after STEMI.

Serum iron and IL-6 concentrations were measured from specimens of venous blood obtained prior to PCI. A second sample was taken after an 8-hour fast to evaluate the lipid profile and measure serum glucose. Diagnosis of STEMI was based on a universal definition of myocardial infarction [17]. Specifically, symptoms of ischemia, ST segment elevation >0.2 mV in ≥2 contiguous electrocardiogram (ECG) leads, and an increase in systemic cardiac biomarkers (for example, troponin I and creatinine kinase (CK) MB mass) with at least one value above the 99^th^ percentile of the upper reference limit within 24 hours of the onset of pain were considered diagnostic of AMI. The culprit vessel was identified based on clinical, ECG and angiographic findings. All patients were administered aspirin and clopidogrel before PCI. Echocardiography was undertaken within the first 2 days after primary PCI and 6 months later. Change in heart function was calculated by subtracting the LV ejection fraction at baseline from ejection fraction at 6 months, divided by baseline ejection fraction. Improvement in heart function was defined as a change in LVEF ≥10%, according to the clinical study results by Ndrepepa et al [18]. Data collected from the subjects included age, sex, and the presence of risk factors (*e.g.*, cigarette smoking, diabetes mellitus, hypertension, and hypercholesterolemia), clinical signs and current medication history. TIMI risk score for STEMI was calculated for each patient based on the description by Morrow et al. [15]. The protocol was approved by the Institutional Review Board of the Changhua Christian Hospital, Taiwan, and all subjects gave written and informed consent to participate.

### Measurement of serum iron and IL-6 concentrations

Serum iron concentration was measured by a timed-endpoint method using a commercially available kit (Synchron Systems FE/IBCT Calibrator Kit; Beckman Coulter, Fullerton, CA, USA), which measures iron bound to transferrin. Serum IL-6 concentration was determined by a commercially available ELISA kit (Human IL-6 ELISA Ready-SET-Go!; eBioscience, San Diego, CA, USA).

### Angiographic assessments

Quantitative coronary angiographic measurements were taken by a cardiologist blinded to the results of other investigations. The TIMI flow [19] and myocardial blush [20] grades for assessing microvascular perfusion were also reviewed by the same cardiologist. Characteristic coronary lesions were classified according to the American College of Cardiologists/American Heart Association classification [21].

### Echocardiographic assessments

We used a modified Simpson’s method to calculate LV ejection fraction as described by the American Echocardiographic Society [22]. Diastolic function was evaluated using Pulsed-wave Doppler recordings. Pulsed wave Doppler measurements were obtained from the apical four-chamber view. Peak E and A wave velocities and E wave deceleration time (DT) were measured from the mitral leaflet tip according to American Society of Echocardiography guidelines [23]. Regional wall motion score index (WMSI) were calculated as the sum of wall motion scores divided by the number of visualized segments (from 17-segment model), where 1 indicates normal; 2, hypokinesis; 3, akinesis; and 4, dyskinesis [24]. After AMI extensive regional wall motion abnormalities may be present but when compensated by regional hyperkinesis of the normal segments, LVEF will be (almost) normal; in these patients, WMSI could more correctly reflect the magnitude of myocardial damage [25].

### Statistical analysis

We performed a sample size calculation according to the results of our pilot study because no case-control trials using the same laboratory test method for serum iron have been reported. A sample size of 19 patients in each group was determined to be sufficient to detect differences in serum iron concentration between the STEMI group and control group, assuming a standard deviation of 37.3, using a two-sided test of the difference between means, a type I error of 5%, and a power of 90%. We expended the sample size to 55 patients in the STEMI group and 54 subjects in the control group to enhance the reliability of the study. Based on current sample size, we had a mean of 62.5 µg/dl of serum iron and a standard deviation of 37.7 µg/dl in the STEMI group, and a mean of 103.0 µg/dl of serum iron and a standard deviation of 38.1 µg/dl in the control group, and then we have a power of 99.99%. We calculated the power from a reliable, freely available website is: http://www.stat.uiowa.edu/~rlenth/Power/index.html [26].

The Mann–Whitney U test was used to examine differences in serum iron concentration between patients whose LV ejection fraction had improved ≥10% over 6 months and those in whom ejection fraction improved <10%. A Spearman’s rho correlation was used to analyze the relationships between serum iron concentration and patient characteristics. A general linear model technique and linear regression were used to evaluate independent associations between serum iron and IL-6 concentrations. The Jonckheere-Terpstra test was used to analyze the association between the serum iron concentration and TIMI risk scores. Trend analysis for IL-6 concentrations after they had been divided into tertile groups was undertaken using the Jonckheere-Terpstra test, which was also used to analyze the association between IL-6 concentrations and serum iron concentrations. The Jonckheere-Terpstra test is similar to the Kruskal-Wallis test but is applied to samples with *a priori* ordering, such as TIMI risk scores, when it has greater statistical power. Parameters showing significant correlations in the univariate analyses were then included in multiple linear regression model to test for significant predictors of improvement in LV ejection fraction 6 months after primary angioplasty. A *P* value<0.05 was considered statistically significant. All statistical analyses were performed on a personal computer with the statistical package SPSS for Windows (Version 15.0, SPSS, Chicago, IL, USA).

## Results

### Baseline serum iron concentration was significantly lower in patients who did not show improvement in LV performance at 6-month follow-up

At 6-month follow-up after PCI, all of 55 AMI patients were alive and there were no adverse cardiac events. We divided the patients into two groups according to the LV performance at 6-month follow-up after PCI. Biochemical and physiological examinations of the patients were summarized in **Table 1**. There were no significant differences between the improvement and non-improvement groups in infarct-related artery location, lesion calcification, lesion complexity, maximal cardiac muscle enzyme concentrations-CKMB, troponin I concentrations, baseline Hb concentrations and 6 month follow-up Hb concentrations, baseline RDW values and 6 month follow-up RDW values, baseline LV ejection fraction, reperfusion quality-post PCI TIMI flow grade, myocardial brush grade and inflammatory marker-IL6. However, serum iron concentration was significantly lower in the non-improvement subgroup (80.8±50.8 *versus* 52.7±24.1 µg/dl, *P* = 0.016).

**Table 1:** Characteristics of patients grouped by change in left ventricular ejection fraction 6 months after percutaneous coronary interventions for acute myocardial infarction.

	Improvement in ejection fraction ≥10%(n = 19)	No improvement in ejection fraction(n = 36)	*P* value
**Sex (M/F)**	14/5	33/3	0.853
**Age, years**	53.3±14.7	59.1±8.9	0.124
**BMI, kg/m^2^**	26.2±3.7	25.3±3.6	0.441
**CPK,** µ**/l (Maximal)**	2101±1817	2410±2140	0.862
**CKMB, ng/ml (Maximal)**	190±173	249±192	0.353
**Troponin I, ng/ml**	4.08±10.58	6.18±18.56	0.928
**Fasting glucose, mg/dl**	146±74	159±93	0.600
**HBA1C, %**	6.0±1.0	6.6±1.8	0.246
**Cholesterol, mg/dl**	193±51	194±46	0.894
**HDL-C, mg/dl**	38.9±9.5	42.9±9.7	0.098
**LDL-C, mg/dl**	134.8±40.9	138.0±39.9	0.614
**Creatinine, mg/dl**	1.01±0.24	1.01±0.36	0.537
**hsCRP, mg/l**	0.56±0.62	0.42±0.73	0.578
**IL-6, pg/ml**	19.8±9.8	16.1±8.5	0.993
**Fibrinogen, mg/dl**	439±102	449±89	0.452
**Serum iron,** µ**g/dl**	80.8±50.8	52.7±24.1	0.016*
**Statin used, %**	80	89	0.324
**TIMI risk score**	2.53±1.20	2.91±1.07	0.191
**LVMI, g/m^2^**	113.3±26.9	109.0±23.9	0.839
**LV EF at baseline, %**	57.8±11.3	62.2±10.3	0.135
**LVEF at 6** **M, %**	67.9±10.1	59.8±11.4	0.005*
**E/A ratio at baseline**	1.07±0.46	1.05±0.46	0.866
**DT at baseline, ms**	162.5±44.3	187.7±53.2	0.104
**E/A ratio at 6** **M**	1.08±0.50	0.93±0.32	0.252
**DT at 6** **M, ms**	199.9±66.7	201.6±52.5	0.422
**EDV at baseline, ml**	80.9±26.4	73.2±17.9	0.396
**ESV at baseline, ml**	35.1±17.4	27.7±11.7	0.222
**WMSI**	1.29±0.21	1.26±0.19	0.730
**Hb, g/dl**	14.7±1.5	14.4±1.5	0.449
**Hb at 6** **M, g/dl**	13.9±2.0	13.8±1.5	0.602
**Hematocrit, %**	42.7±4.4	53.5±7.7	0.410
**Hematocrit at 6** **M, %**	40.5±6.0	40.9±3.9	0.759
**RDW, %**	13.58±0.74	13.79±0.84	0.416
**RDW at 6** **M, %**	13.56±0.74	14.20±1.17	0.102
**Culprit lesion**	LAD (10)	LAD (20)	0.992
	LCX (3)	LCX (3)	
	RCA (6)	RCA (13)	
**D2B time, minutes**	85.5±38.4	84.3±38.0	0.916
**Myocardial brush grade**	2.10±0.87	2.00±0.93	0.693
**Lesion length (mm)**	19.8±7.9	19.1±5.8	0.726
**Pre-PCI TIMI flow grade**	0.35±0.79	0.88±1.20	0.079
**Post-PCI TIMI flow grade**	2.97±0.18	3.00±0.00	0.384
**Lesion calcification**	0.26±0.57	0.13±0.44	0.355
**Lesion complexity**	1.03±0.40	0.88±6.12	0.284

*P<0.05, Mann–Whitney U test.

6 M, six months; BMI, body mass index; CPK, creatine- phosphor-kinase; CKMB, creatine phosphokinase-MB; HbA1C, glycohemoglobin; HDL-C, high density lipoprotein cholesterol; LDL-C, low density lipoprotein cholesterol; hsCRP, high sensitivity C reactive protein; IL-6, interleukin 6; WMSI, wall motion score index; TIMI, thrombolysis in myocardial infarction; LVMI, left ventricular mass index; LVEF, left ventricular ejection fraction; E/A ratio, the ratio of the peak velocities of early (E wave) and late (A wave) diastolic filling; DT, the deceleration time of the E wave; EDV, left ventricular end diastolic volume; ESV, left ventricular end systolic volume; Hb, hemoglobin; RDW, red blood cell distribution width; LAD, left anterior descending artery; RCA, right coronary artery; LCX, left circumflex artery; D2B, door to balloon; PCI, percutaneous coronary interventions; TIMI, thrombolysis in myocardial infarction.

### Factors correlated with serum iron concentration after AMI

Hb concentration, hematocrit, lymphocyte counts, the proportion of patients taking statins, and LV ejection fraction at 6 months were positively correlated with serum iron concentration (**Table 2**); however, TIMI risk score was negatively correlated with serum iron concentration.

**Table 2:** Univariate correlation between serum iron concentration and patient characteristics after acute myocardial infarction.

Variable	*Rho* correlation coefficient	*P* value
**Hemoglobin, g/dl**	0.273	0.036*
**Hematocrit, %**	0.257	0.047*
**Lymphocyte, %**	0.276	0.035*
**Statin used**	0.273	0.028*
**TIMI risk score**	−0.346	0.009*
**EF 6** **M, %**	0.301	0.025*

*P<0.05, Spearman’s rho correlation.6 M, six months; TIMI, thrombolysis in myocardial infarction; EF 6 M, left ventricular ejection fraction at 6–month follow-up.

### Serum iron concentration significantly decreased as TIMI risk score increased after primary angioplasty

**Figure 1:**
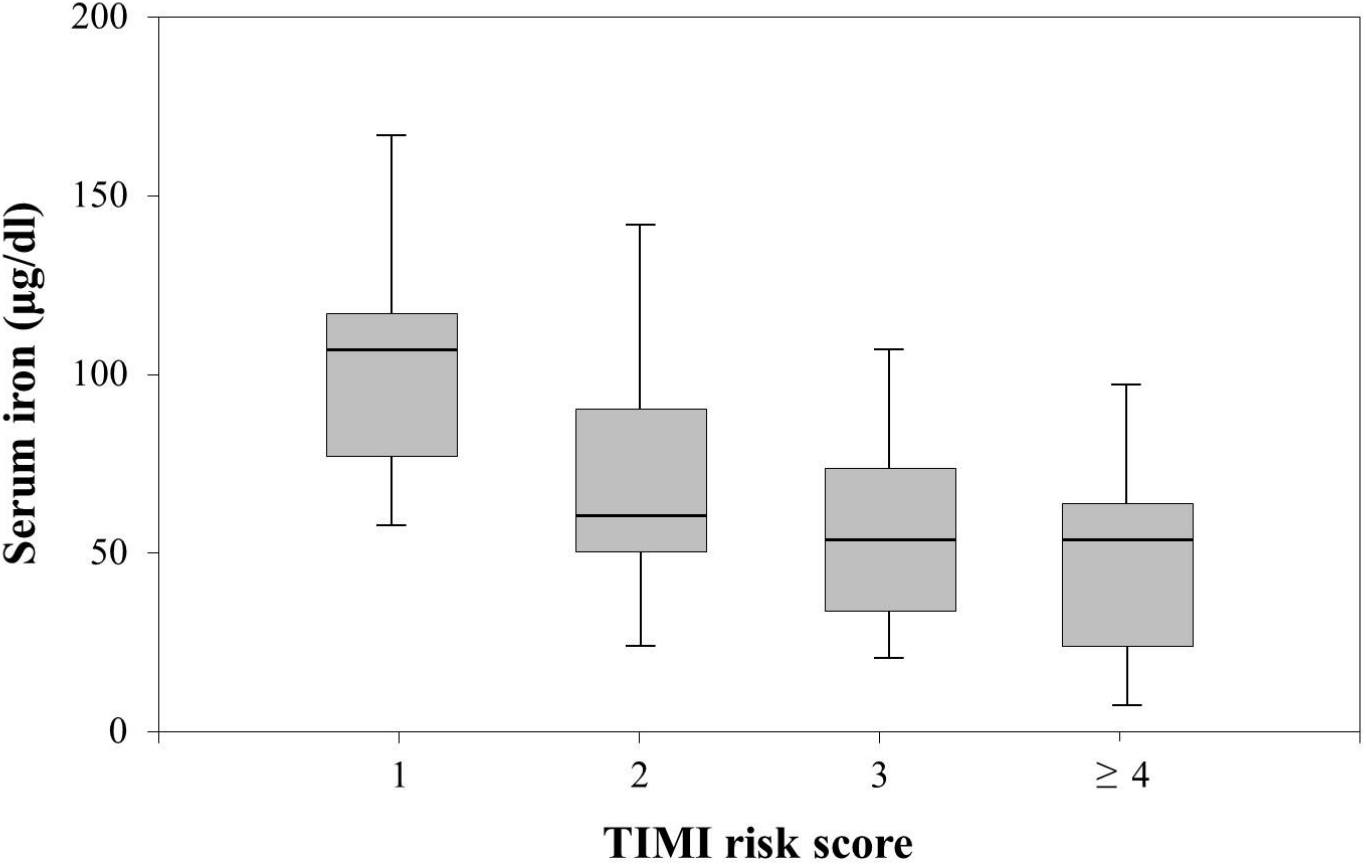
The relationships between serum iron concentration and TIMI risk scores after primary angioplasty for AMI. The AMI patients were divided into four subgroups according to TIMI risk score for STEMI: Group 1 (TIMI risk score 1, n = 8); Group 2 (TIMI risk score 2, n = 15); Group 3 (TIMI risk score 3, n = 19); and Group 4 (TIMI risk score ≥4, n = 13). Trend analysis with Jonckheere-Terpstra test found that serum iron concentration significantly decreased as TIMI risk score rose (*P* = 0.002).

We divided AMI patients into four subgroups according to TIMI risk score for STEMI: group 1, TIMI risk score 1 (n = 8); group 2, risk score 2 (n = 15); group 3, risk score 3 (n = 19); and group 4, risk score ≥4 (n = 13). Trend analysis found that serum iron concentration significantly decreased as TIMI risk score rose (**Figure 1**; Jonckheere-Terpstra test, *P* = 0.002).

### The relationship between serum iron and interleukin-6 levels in all study subjects

**Figure 2:**
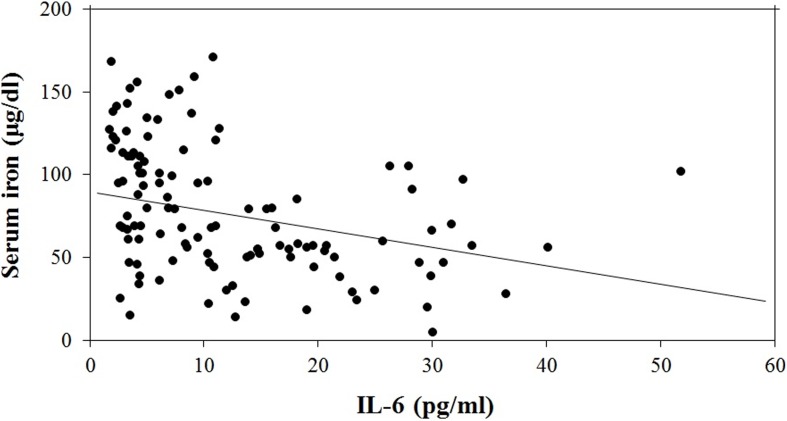
The relationship between serum iron concentration and IL-6 levels in all enrolled subjects. The result indicated that the serum iron concentration was negatively correlated with circulating IL-6 concentration in all study subjects. The linear relationship was well described by Serum iron = 95.994−1.246 (IL-6), R^2^ = 0.133 and *P*<0.001.

Serum iron concentration in the AMI group was significantly lower than in the control group (62.5±37.7 *vs.* 103.0±38.1 µg/dl, *P*<0.001) and IL-6 concentration was significantly higher in the AMI group than in the control group (17.80±13.19 *vs.* 6.98±8.26 pg/ml, *P*<0.001). Linear regression model was used to evaluate the independent associations between serum iron and IL-6 concentrations in all enrolled subjects. We found that serum iron concentration was negatively correlated with circulating IL-6 concentration (**Figure 2**; Serum iron = 95.994−1.246 (IL-6), R^2^ = 0.133, *P*<0.001). We found no correlation between IL-6 and Hb concentration in all study subjects. Furthermore, we found that serum iron concentration in the control group was, on average, 35.494 µg/dl higher than that in the AMI group. For every one unit increase in IL-6 concentration, there was a decrease of 0.625 units in serum iron concentration (**Table 3**).

**Table 3:** The relationship between serum iron and interleukin-6 levels in all subjects.

Parameter	Estimate	SE	95% Confidence interval	*P* value
**Intercept**	71.872	6.984	58.040–85.704	<0.001**
**Group (Control vs. AMI)**	35.494	7.440	20.759–50.229	<0.001**
**IL-6**	−0.625	0.299	−1.218−−0.032	0.039*

(Dependent variable: serum iron concentration).

*P<0.05 and **P<0.001, general linear model. AMI, acute myocardial infarction; IL-6, interleukin 6; SE, standardized error. The intercept is the predicted value of serum iron concentration in AMI group. The predicted value is 71.872 µg/dl. The serum iron concentration in the control group was, on average, 35.494 µg/dl higher than that in the AMI group. For every one unit increase in IL-6 concentration, there was a decrease of 0.625 units in serum iron concentration.

### Trend analysis showed serum iron concentration, not Hb, was inversely proportional to IL-6 concentration

**Figure 3:**
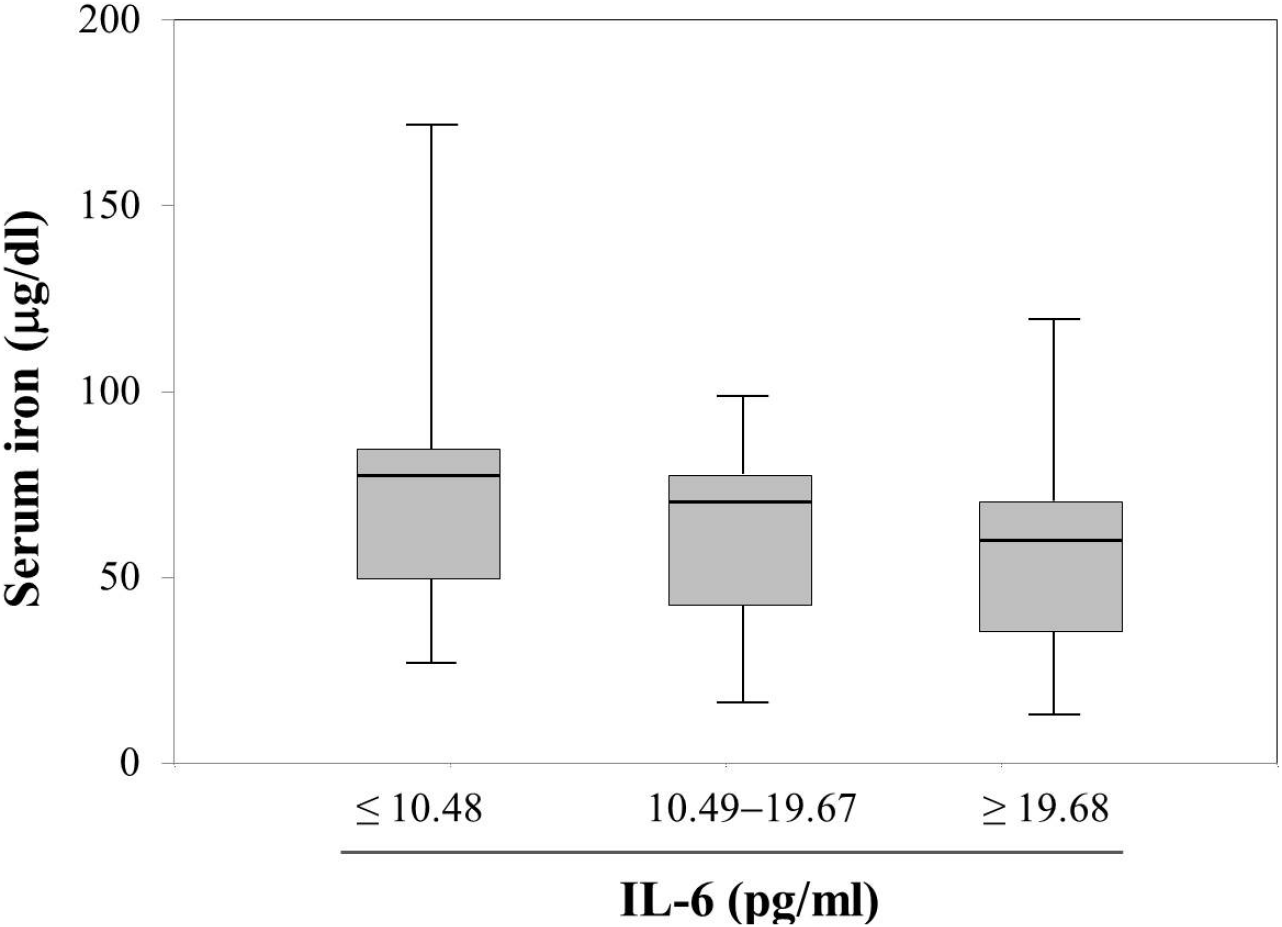
Trend analysis showed serum iron concentration was inversely proportional to IL-6 concentration in STEMI patients. AMI patients were divided into three subgroups according to circulating IL-6 concentration tertile: group 1, IL-6 concentration ≤10.48 pg/ml (n = 19); group 2, IL-6 concentration between 10.49–19.67 pg/ml (n = 20); group 3, IL-6 concentration ≥19.68 pg/ml (n = 16). Trend analysis showed serum iron concentration was inversely proportional to IL-6 concentration. (Jonckheere-Terpstra test, *P* = 0.043).

We divided AMI patients into three subgroups according to circulating IL-6 concentration tertile: group 1, IL-6 concentration ≤10.48 pg/ml (n = 19); group 2, IL-6 concentration between 10.49–19.67 pg/ml (n = 20); group 3, IL-6 concentration ≥19.68 pg/ml (n = 16). Trend analysis found that serum iron concentration significantly decreased as serum IL-6 concentration rose (**Figure 3**; Jonckheere-Terpstra test, *P* = 0.043).

### Lower serum iron concentration is associated with higher TIMI risk score and has higher inflammatory markers after AMI

The patients in AMI group was divided into two subgroups, lower serum iron (<60 µg/dl) and higher serum iron (≥60 µg/dl), on the basis of the median serum iron concentration (**Table 4**). Patients in the lower serum iron subset had significantly higher concentrations of fibrinogen and IL-6 (*P*<0.05). Both fibrinogen and IL-6 are biomarkers of inflammation related with cardiovascular diseases. It is also noted that patients in the lower serum iron subset showed a higher TIMI risk score after AMI (*P*<0.05).

**Table 4:** Lower serum iron concentration is associated with higher inflammatory markers and TIMI risk score after acute myocardial infarction.

	Lower serum iron (<60 µg/dl)	Higher serum iron (≥ 60 µg/dl)	*P* value
	n = 34	n = 21	
**IL-6, pg/ml**	19.8±14.6	14.2±9.4	0.042*
**Fibrinogen, mg/dl**	470±93	421±86	0.040*
**TIMI risk score**	3.10±1.00	2.43±1.27	0.025*

*P<0.05, Mann–Whitney U test. TIMI, thrombolysis in myocardial infarction; IL-6, interleukin 6.

### Baseline serum iron level predicts LV function 6 months after primary angioplasty for AMI

Multiple linear regression analysis of variables associated with LV performance 6 months after primary angioplasty was undertaken. The following variables were entered into the model: serum iron, IL-6, infarct related artery location, peak creatine kinase-MB and WMSI. It showed that serum iron concentration at the time of AMI could predict LV ejection fraction 6 months later, even when traditional prognostic factors such as LV wall motion score index, cardiac enzyme and IL-6 concentrations, and infarct locations were taken into account (**Table 5**). Baseline Hb concentrations, follow-up Hb concentrations, baseline RDW values and follow-up RDW values did not predict LV ejection fraction at 6 months when the same prognostic factors were considered.

**Table 5:** Multiple linear regression analysis of variables associated with ejection fraction 6 months after primary angioplasty for acute myocardial infarction.

Explanatory variable	Unstandardized Coefficients		Standardized Coefficients	t	*P* value
	B	Std. error	Beta		
**(Constant)**	106.249	7.684		13.833	0.000
**Serum iron**	0.069	0.029	0.227	2.415	0.020*
**CPK MB**	−0.012	0.006	−0.200	−1.965	0.055
**IL-6**	−0.006	0.082	−0.007	−0.068	0.946
**WMSI**	−36.606	6.510	−0.631	−5.723	<0.001**
**IRA (LAD ** ***vs.*** ** RCA)**	0.943	2.670	0.041	0.353	0.725
**IRA (LCX ** ***vs.*** ** RCA)**	9.890	3.825	0.269	2.585	0.013*

(Dependent variable: ejection fraction at 6 months).

*R^2^* = 0.620.

*P<0.05 and **P<0.001.

CPK MB, creatine phosphokinase-MB; IL-6, interleukin 6; WMSI, wall motion score index; IRA, infarct related artery; LAD, left anterior descending artery; RCA, right coronary artery; LCX, left circumflex artery.

## Discussion

Our findings confirm the important role of serum iron in determining LV performance after STEMI. Furthermore, the recovery of LV function was impaired in those with lower serum iron concentration after revascularization. Anemia can be viewed as the net result of iron deficiency, but a normal Hb level does not necessarily mean that iron stores are adequate. An individual with normal body iron stores must lose a large portion of body iron before the Hb falls below the laboratory definition of anemia (generally, Hb<12 g/dl for women and Hb<13 g/dl for men). To investigate whether serum iron or Hb or RDW is the most important determinant of LV function after STEMI, multiple regression analyses were performed with serum iron or Hb concentration or RDW value as one of the covariates. In these analyses, serum iron concentrations were significantly predictive of LV systolic function after adjusting for possible confounding factors (such as infarct location, LV wall motion score index, and serum concentrations of cardiac enzymes and IL-6). Blood Hb concentrations and RDW values at baseline and at 6 months were found not to be significant predictors when the same prognostic factors were considered. These findings suggest that serum iron concentration is an independent predictor of LV systolic function after STEMI, rather than Hb level or RDW value. This conclusion is supported by a previous study that showed that the symptoms and signs of CHF can be ameliorated by the administration of intravenous iron regardless of whether the patient is anemic or not [2]. Furthermore, administration of an iron chelator after AMI but before PCI does not appear to get clinical benefit [27]. In addition, lower serum iron concentrations are associated with higher concentrations of proinflammatory markers and lower concentrations of the cardioprotectant insulin-like growth factor-1 in ischemic heart disease [28]. These data support the concept that serum ion is tightly regulated.

Iron is an essential micronutrient as it is required for satisfactory erythropoietic function, oxidative metabolism and cellular immune response. Functionally, iron exists in the body in two pools: utilized and stored [1]. Utilized iron consists of circulating and intracellular iron. Iron released into the circulation binds to transferrin and is transported to sites of use and storage [29]. The amount of transferrin-bound iron is around 4 mg, but this is the most important functional dynamic iron pool [29]. The vast majority of intracellular iron is in erythrocyte Hb and circulating reticulocytes. Iron pools interact with each other, and iron can be transferred between these compartments using tightly regulated mechanisms. A normal Hb level with a mean corpuscular Hb (MCH) in the lower limit of the normal range (28–35 pg) or an increased red cell distribution width (RDW, normal range 11–15%) indicates mild iron deficiency without anemia. Although a rising RDW may be an earliest indicator of iron deficiency, the main laboratory finding is a low serum ferritin concentration [1]. However, an RDW value within the reference interval can be used to exclude iron deficiency in those cases in which the serum ferritin concentration does not accurately reflect the iron stores owing to severe tissue damage, as in inflammation [30]. This is the reason why we did not check serum ferritin in STEMI. We also did not measure total iron-binding capacity (TIBC); hence, we were unable to determine participants’ complete iron status. However, indirect data suggest that our subjects had normal iron stores, reflected in largely normal MCH and RDW values. Conceptually, a low serum iron concentration might be indicative of a diminished utilized iron pool. Cells with a high mitogenic potential (neoplastic, hematopoietic, immune) [31], [32] and high-energy demand (hepatocytes, adipocytes, skeletal and cardiac myocytes, and renal cells) [33], [34] are particularly sensitive to depleted iron supplies and/or abnormal iron utilization.

ACS is a disease characterized by inflammation-induced atherosclerotic plaque rupture, subsequent thrombus formation, coronary artery occlusion, and myocardial oxygen deficiency [35]. Hypoferremia is a common response to systemic infections or generalized inflammatory disorders. A recent longitudinal study demonstrated that IL-6 was a better predictor of CHF after ACS than CRP [13]. Our findings are in broad agreement with a previous report that elevated IL-6 concentrations immediately after AMI were negatively correlated with LV ejection fraction 6 months later [36]. We demonstrated clearly that serum iron concentration was inversely proportional to IL-6 concentration: For every one unit increase in IL-6 concentration, there was a decrease of 0.625 units in serum iron concentration. We also found that serum iron concentration in the control group was on average 35.494 µg/dl higher than that in the AMI group. However, there was no correlation between Hb concentrations and IL-6 concentration. One previous study conducted in human liver cell culture, mice and human volunteers reported that IL-6 was necessary and sufficient for the induction of hepcidin during inflammation, and that the IL-6–hepcidin axis was responsible for inflammation-induced hypoferremia [37]. In rodents [38] and humans [39], AMI was accompanied by increased circulating hepcidin, which subsequently subsided during recovery. These findings led us to postulate that after AMI, circulating IL-6 level increases, followed by elevation in hepcidin and a decrease in serum iron concentrations. However, reduced serum iron concentration represents depletion of the main functional iron pool, which could modulate LV function. Our hypothesis is also supported by the finding that after AMI, increased circulating IL-6 concentrations at baseline and after hospital discharge were associated with heart failure and impaired LV ejection fraction [40]. Low serum iron concentration may explain why activation of proinflammatory factors is believed to enhance myocardial damage, leading to dysfunction and heart failure.

The novelty of our study is that we analyzed the relationship between serum iron concentration and TIMI risk score for STEMI. In patients with ACS, risk scores are useful short- and long-term predictors of death and cardiovascular complications [15], [16]. The score is calculated from patients’ age, body mass, blood pressure and heart rate on admission and the Killip class. It was significantly higher in patients with low baseline serum iron concentration and *vice versa*, suggesting that serum iron has a protective effect on myocardial function.

The major limitation of the study is that it does not provide mechanistic insight. Other limitations include the relatively small size of the cohort, and the fact that iron status markers were only measured at baseline. Therefore, larger studies of patients with STEMI that measure serum iron concentration, total iron binding capacity and ferritin are needed to further confirm our hypothesis. These might form the basis of intervention studies, to examine whether iron supplementation and/or control of serum IL-6 concentration might have a beneficial effect on the recovery of LV function after STEMI.

## Conclusions

We found an association between lower serum iron concentration before PCI and impaired recovery of LV systolic function 6 months later. The circulating concentration of IL-6 was increased after STEMI and negatively correlated with serum iron concentration. Serum iron concentration was also negatively correlated with TIMI risk score. Our findings support the hypothesis that higher serum iron concentration is associated with a cardioprotective phenotype, and that it is a potential prognostic biomarker for complications after STEMI, and may be a novel biomarker that could therapeutic intervention.
